# Sex differences and the effect of female sex hormones on auditory function: a systematic review

**DOI:** 10.3389/fnhum.2023.1077409

**Published:** 2023-04-21

**Authors:** Nada Aloufi, Antje Heinrich, Kay Marshall, Karolina Kluk

**Affiliations:** ^1^Manchester Centre for Audiology and Deafness, Manchester Academic Health Science Centre, Faculty of Biology, Medicine and Health, School of Health Sciences, University of Manchester, Manchester, United Kingdom; ^2^College of Medical Rehabilitation Sciences, Taibah University, Medina, Saudi Arabia; ^3^Division of Pharmacy and Optometry, Faculty of Biology, School of Health Sciences, Medicine and Health, University of Manchester, Manchester, United Kingdom

**Keywords:** sex differences, female hormones, auditory function, fluctuation, progesterone, estradiol

## Abstract

**Aims:**

First, to discuss sex differences in auditory function between women and men, and whether cyclic fluctuations in levels of female sex hormones (i.e., estradiol and progesterone) affect auditory function in pre-menopausal and post-menopausal women. Second, to systematically review the literature concerning the discussed patterns in order to give an overview of the methodologies used in research. Last, to identify the gap in knowledge and to make recommendations for future work.

**Methods for the systematic review:**

Population, Exposure, Control, Outcome and Study design (PECOS) criteria were used in developing the review questions. The review protocol follows the guidelines of the Preferred Reporting Items for Systematic Reviews and Meta-Analyses (PRISMA) and was pre-registered in the Prospective Register of Systematic Reviews (PROSPERO; CRD42020201480). Data Sources: EMBASE, PubMed, MEDLINE (Ovid), PsycINFO, ComDisDome, CINAHL, Web of Science, Cochrane Central Register of Controlled Trials (CENTRAL) via Cochrane Library, and scanning reference lists of relevant studies, and internet resources (i.e., Mendeley) were used. Only studies published between 1999 and 2022, in English, or in English translation, were included. The quality of evidence was assessed using the Newcastle-Ottawa Scale (NOS).

**Results:**

Sex differences: Women had more sensitive hearing (measured at the level of peripheral and central auditory system) than men. Cyclic fluctuations: Auditory function in women fluctuated during the menstrual cycle, while no such fluctuations in men over the same time period were reported. Hearing sensitivity improved in women during the late follicular phase, and decrease during the luteal phase, implying an effect of female sex hormones, although the specific effects of estradiol and progesterone fluctuations on the central auditory system remain unclear. Hearing sensitivity in women declined rapidly at the onset of menopause.

**Conclusion:**

The review has shown the following. Consistent sex differences exist in auditory function across the auditory pathway with pre-menopausal women often showing better function than age-matched men. Moreover, pre-menopausal women show fluctuations in hearing function across the menstrual cycle with a better function during the peak of estradiol or when the ratio of estradiol to progesterone is high. Third, menopause marks the onset of hearing loss in women, characterized by a rapid decline in hearing sensitivity and a more pronounced loss than in age-matched men. Finally, the systematic review highlights the need for well-designed and -controlled studies to evaluate the influence of estradiol and progesterone on hearing by consistently including control groups (e.g., age-matched man), using objective tests to measure hormonal levels (e.g., in saliva or blood), and by testing participants at different points across the menstrual cycle.

**Systematic review registration:**

https://www.crd.york.ac.uk/prospero/display_record.php?ID=CRD42020201480, identifier CRD42020201480.

## Definitions

**Table:** 

Pre-menopause	The reproductive period of a woman’s life.
Menopause	A time period of 12 consecutive months after the cessation of a woman’s menstrual cycle.
Post-menopause	The time period after menopause, extended time period of amenorrhea.
Amenorrhea	The absence of menstruation.
Menstrual cycle phases	Phases of menstrual cycle are defined in this review relative to a typical 28-day cycle (in shorter cycles the follicular phase is attenuated and elongated in longer cycles) and are described as: •*Early Follicular phase:* day 1–8 of the cycle, where day 1 is the first day of menses (start of menstrual cycle) •*Late Follicular phase:* day 9–16 of the cycle (day 14 – ovulation) •*Early Luteal phase:* day 17–22 of the cycle •*Late Luteal phase:* day 23–28 of the cycle
Pure Tone Audiometry (PTA)	Behavioral test used to assess hearing sensitivity. Typically measured at 0.25, 0.5, 1, 2, 4, 6, and 8 kHz.
Oto-Acoustic Emissions test (OAEs)	Physiological test used to assess the health of outer hair cells (OHCs) in the cochlea by recording soft sounds emitted by the ear. OAEs can be spontaneous, i.e., Spontaneous Oto-Acoustic Emissions (SOAEs), and evoked by a click/tone burst (Transient Evoked Oto-Acoustic Emissions, TEOAESs), or by a combination of two tones (Distortion Product Oto-Acoustic Emissions (DPOAEs).
Auditory Brainstem Response (ABR)	Physiological measure of auditory pathway’s neuroelectric activity from the auditory nerve to the cerebral cortex. ABRs can be evoked by a range of stimuli such as clicks, tone-bursts or complex stimuli.
Speech – ABR	ABR evoked by speech (e.g.,/ba/). Speech-ABR provides critical information on how more complex stimuli are processed by the brainstem.
Speech audiometry	Behavioral test used to assess speech recognition threshold (SRT), i.e., at what sound level does the speech need to be presented to be accurately perceived in fifty percent of the cases; and word recognition score (WRS), i.e., what proportion of words is accurately perceived at a particular presentation level of sound. Additional to speech perception, speech discrimination and comprehension can be tested to assess the ability to discriminate between similar words and comprehend sentences and continuous speech.
Women	Women are defined in this review as an adult who was identified as female at birth. This was chosen as most of the literature reviewed was published before the definition of the word “women” in Cambridge Dictionary was expanded (October 2022) to include an adult who lives and identifies as female though they may have been identified with a different sex at birth. Consequently, in this review the words “female” and “women” have the same definitions.
Men	Men are defined in this review as an adult who was identified as male at birth. This was chosen as most of the literature reviewed was published before the definition of the word “men” in Cambridge Dictionary was expanded (October 2022) to include an adult who lives and identifies as a male though they may have been identified with a different sex at birth. Consequently, in this review the words “male” and “men” have the same definitions.

## 1. Introduction

This is a narrative review followed by a systematic review of the available evidence on sex differences in auditory function, and the effect of changes in female sex hormone levels on hearing. By identifying these sex differences, researchers and clinicians will be able to understand the static impact of sex on different audiometric measures, and impact of dynamic fluctuations in sex hormones on hearing function. In addition, this review highlights the methodological concerns in research studies investigating sex differences and/or the effect of sex hormones on hearing. This can be used to improve future work in this field. Lastly, this review highlights the questions for which the available evidence provides a clear possibility of hormonal treatment for preserving hearing sensitivity in older women.

Sex differences in hearing have been reported by some (McFadden, 1993; Stuart and Kerls, 2018; Zakaria et al., 2019) but not others (Wadnerkar et al., 2008; Boothalingam et al., 2018). It is not clear whether these differences are genuine and occur due to biological sex differences (such as differences in sex hormones) or whether they are due to systematic differences between the sexes in exposure to environmental noise and/or ototoxins. Three pieces of evidence support the hypothesis that these differences are due to biological differences and that female sex hormones contribute to sex differences in hearing. Firstly, Turner syndrome patients (young women with abnormally low levels of female sex hormones) present with hearing thresholds comparable to those of women in control population at least 20 years older than their age group (Bonnard et al., 2017, 2018), which points to a protective role of sex hormones. Secondly, better hearing sensitivity in young women compared to age-matched men (McFadden et al., 2006) disappears, i.e., hearing sensitivity in women decreases, when women reach menopause (reduction in female sex hormones). Thirdly, women’s hearing function fluctuates cyclically in synchrony with fluctuations in female sex hormones (Al-Mana et al., 2010). Moreover, biological sex has been reported to be associated with some aspects of cochlear function and its vulnerability to changes due to age or noise exposure. This is in particular due to the protecting mechanism of female sex hormones against noise exposure, and delaying the onset of age-related hearing loss in women (McFadden, 1998; Zündorf et al., 2011; Grinn et al., 2017; Shuster et al., 2019). In the following sections we will review first sex hormones in women and men, followed by description of auditory anatomy, functioning of the relevant sex hormones, and finish with a discussion of evidence for and against static and dynamic differences in hearing associated with sex hormones. We will then systematically review the literature to provide an overview of the methods and outcome measures used in the field. Finally we will summarize gaps of knowledge in the field and suggest potential ways forward.

### 1.1. Sex hormones

Hormone status differs between women and men during the reproductive years of life, both in the overall levels of hormones and in terms of regular fluctuations over time. In general, similar sex hormones (i.e., estradiol, progesterone, and testosterone) can be found both in women and men, however, the production sites, blood concertation, and their effect on different organs and systems differ greatly (Svechnikov and Söder, 2007). In women, estradiol (the most potent of the three naturally occurring estrogens) and progesterone are secreted by the ovaries in a cyclic pattern of high/low amounts (across the reproductive cycle), while testosterone is produced only in small amounts by both ovaries and the adrenal glands (Svechnikov and Söder, 2007). In men, high amounts of testosterone are secreted by the testes, while small amounts of estradiol and progesterone are produced by both the testes and adrenal glands (Tyagi et al., 2017). In men, hormone levels are relatively stable (Lauretta et al., 2018), while in women hormone levels fluctuate across the reproductive cycle and change across the lifespan. Estradiol is made in the adrenal glands, ovaries, and fat cells, and is found in both sexes, but its concentration in blood is higher in women than men. While the levels of estradiol fluctuate during the different stages of a woman’s life (i.e., menstrual cycle, during pregnancy, and menopause), in men the level of this hormone remains largely stable (Lauretta et al., 2018). Progesterone, which is produced by the corpus luteum (He and Ren, 2018), counters the function of estradiol in non-pregnant women. It is mainly responsible for stimulating the ovaries to develop a new menstrual cycle and preparing the endometrium for implantation of the fertilized egg, thus its levels rise in the luteal phase (Simonoska et al., 2009). Progesterone is also the dominant hormone during pregnancy, as the placenta takes over the function of corpus luteum to secrete progesterone (He and Ren, 2018). Prolonged changes in hormone status, for instance during pregnancy when progesterone dominates, or menopause when overall sex hormone levels decline, have been associated with reduced hearing sensitivity (e.g., Guimaraes et al., 2006; Al-Mana et al., 2008; Emami et al., 2018).

The reproductive time span in women begins at menarche (pre-menopausal) and ends when the menstrual cycle ceases (amenorrhea) and a woman enters a period called menopause. When amenorrhea lasts for longer than 12 consecutive months, a woman enters a period called post-menopause. There are distinct hormonal changes that coincide with these three phases of a non-pregnant woman’s reproductive cycle: In pre-menopausal period, the amount of female sex hormones (e.g., estradiol and progesterone) fluctuates cyclically during the menstrual cycle.

The following section outlines the cyclical characteristics of the ovarian cycle and is followed by a discussion of the two hormones with particular relevance to hearing: estradiol and progesterone.

#### 1.1.1. The ovarian cycle

Hormonal regulation in both women and men is controlled by the hypothalamus and the pituitary gland. The main difference in hormonal regulation between the sexes is the frequency of change, i.e., women go through a full female reproductive cycle each month in addition to changes that occur across the lifespan, while in men hormonal changes occur only across the lifespan. The hypothalamus in the female brain produces the gonadotropin-releasing hormone (GnRH), which causes the anterior pituitary gland to produce two hormones (gonadotrophins) that are essential to the ovarian cycle: follicle stimulating hormone (FSH) and luteinizing hormone (LH) (Hawkins and Matzuk, 2008). The concentration of these hormones fluctuates during the menstrual cycle as estradiol has a feedback action upon their release. In most of the cycle, estradiol exerts homeostatic negative feedback on GnRH (Moenter et al., 2009).

**FIGURE 1:**
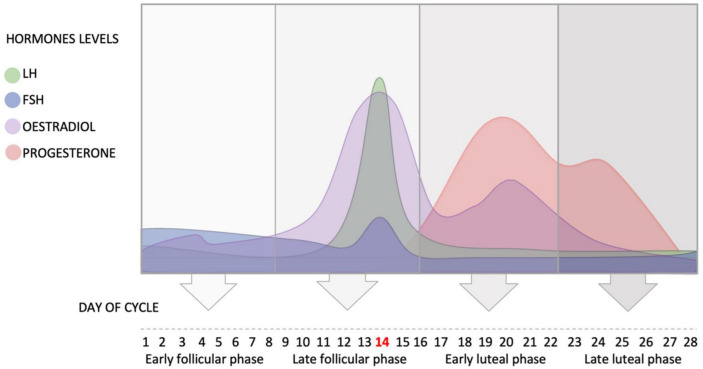
Schematic representation of the fluctuation of the hypothalamus and ovarian hormones during the average ovarian cycle, and the four phases of the cycle. FSH, follicle stimulating hormone; LH, luteinizing hormone.

The average length of the cycle is 28 days (Najmabadi et al., 2020), and it can be divided into four phases: early follicular phase defined as day 1–8 of the cycle, where day 1 is the first day of menses (start of menstrual cycle); late follicular phase defined as day 9–16 of the cycle (day 14 – ovulation); early luteal phase defined as day 17–22 of the cycle; and late luteal phase defined as day 23–28 of the cycle (see Figure 1). Ovulation typically finishes by Day 16 (i.e., 12–14 days before the next menstrual cycle begins). These phases are dominated by different hormones, two of which are of particular interest to hearing: estradiol and progesterone.

In the beginning of the menstrual cycle (early follicular phase), the concentration of estradiol is low. This low concentration of estradiol inhibits the secretion of LH and slightly increases the release of FSH (see Figure 1).

At the end of the early follicular phase, estradiol levels rise leading to positive feedback and the release of GnRH, which in turn activates LH and FSH to surge and initiate ovulation. The pulsatile nature of the release of GnRH determines the ratio of release of the two gonadotrophins. The level of estradiol fluctuates during the menstrual cycle and reaches its peak in the late follicular phase when FSH enters the ovary and helps the primary follicle to develop into a secondary follicle. High levels of estradiol cause positive feedback and the release of LH, which consequently increases its secretion. The high level of LH triggers ovulation and the release of the mature follicle (usually at day 14 in a 28-day cycle).

After ovulation (day 14), the early luteal phase begins in which the level of LH drops dramatically as the level of estradiol decreases (Hawkins and Matzuk, 2008). The corpus luteum (i.e., the remains of the follicle) produces estradiol and progesterone, and progesterone level starts to increase in this phase until it reaches its peak around day 21 (Hawkins and Matzuk, 2008; see Figure 1).

During the early and late luteal phases, progesterone plays an important role in inhibiting the secretion of GnRH in the hypothalamus, in order to prevent the release of FSH and LH and stop the development of a new cycle. Therefore, the levels of GnRH, FSH and LH decrease in the early and late luteal phases (Hawkins and Matzuk, 2008).

The levels of these sex hormones can be assessed using different methods. The most accurate measures are biological measures such as blood or saliva samples where hormone levels are directly assessed. Alternatively, self-report measures can be used, however, this might not be an accurate or consistent measure (Farrar et al., 2015).

### 1.2. Auditory pathway

The human auditory system comprises the peripheral auditory pathways: the external, middle, inner ears, and the vestibulocochlear nerve (8th cranial nerve), which connects to the central nervous system (McFadden, 1998; Zündorf et al., 2011; Grinn et al., 2017; Shuster et al., 2019) and central auditory pathways: cochlear nuclei, superior olivary nuclei, lateral lemniscus, inferior colliculus, medial geniculate nuclei, and auditory cortex (Shuster et al., 2019). Function of the auditory system can be assessed using either behavioral or physiological measures.

#### 1.2.1. Behavioral measures of the auditory function

Behavioral methods require participants’ active cooperation to provide responses. For example, Pure Tone Audiometry (PTA) is a behavioral hearing sensitivity measure that requires participants to indicate (by pressing a response button) when they heard the test sound. Speech Audiometry tests require participants to repeat speech samples that they heard. They provide information on how well the auditory system processes speech signals, which are more similar to natural signals heard in daily life than the pure tones used in PTA. Speech audiometry can be carried out for speech presented in quiet or in background noise. The latter can be useful for assessing not only auditory function but also cortical speech and language function.

#### 1.2.2. Physiological measures of auditory function

Physiological responses are recorded without the need for participants’ active cooperation. For example, Otoacoustic Emissions (OAEs), a marker of the health of the outer hair cells (OHCs) in the cochlea, are recorded from the participants’ ears without any need for participants’ active cooperation (Gold, 1948; Kemp, 1978; Gelfand, 2004; Grabham et al., 2013). Different types of OAEs can be recorded that reflect slightly different aspects of function of OHCs in the cochlea (e.g., Robinette and Glattke, 1997; Grabham et al., 2013). Spontaneous Otoacoustic Emission (SOAE) is a constant unprompted sound emitted from the cochlea that is always present, without any external stimulus. The sound pressure levels of SOAE range between 10 and 30 dBSPL, i.e., they are not usually audible to those who have them (Kemp, 2002). SOAEs are one sign of a healthy cochlea, are spontaneously produced, and are present in 30% (Robinette and Glattke, 1997) to 70% (Abdala and Visser-Dumont, 2001) of all listeners with normal hearing. In contrast, Transient Evoked Otoacoustic Emissions (TEOAEs) are evoked OAEs and can therefore be easily elicited from all healthy ears. TEOAEs are evoked by a short click stimulus and emit complex signals back to the external auditory meatus milliseconds after its presentation. Distortion Product Otoacoustic Emissions (DPOAE) are a third type of OAEs that can be used in order to assess the health of the cochlea. They are evoked when two tones of different frequencies (*f*_1_ and *f*_2_) are presented to the ear and the ear emits back distortion products of the presented sounds. The DPOAE that can be detected most prominently occurs at a frequency equal to *2f_1_–f_2_* of the presented sound.

Auditory Brainstem Responses (ABRs) are electrophysiological measures of hearing sensitivity and auditory function up to the level of brainstem, do not require patient’s cooperation, and are often measured while the patient is asleep (Corwin et al., 1982). ABRs consist of five waves, with each wave originating from a different part of the central auditory system, starting with the spiral ganglion in the cochlea (wave I) all the way to the inferior colliculus (wave V) (McFadden, 1998).

### 1.3. Anatomical evidence for the influence of sex hormones on hearing

#### 1.3.1. Estradiol (E2, or 17β-estradiol) receptors

The following section will discuss anatomical evidence showing that hearing function can be affected by sex hormones. As a first step it is important to note that estradiol has impact beyond the reproductive system and influences the physiological function of other body organs and systems such as the skeletal, cardiovascular, and nervous systems (Al-Mana et al., 2008; He and Ren, 2018). In hearing, estradiol may improve the inflow of metabolites to inner ear cells, which is vital for processing of auditory signals, and has been found to act as a neuromodulator in facilitating detection of auditory signals (Tremere et al., 2009).

Estradiol receptors (ERs) have been found in the inner ear of both animals and humans. The role of ERs is to mediate the effect of estradiol in the cells. Two types of intracellular estradiol receptor exist, ERα and ERβ. ERα are likely to influence the cochlear and vestibular sensory transduction, while ERβ may have more central, neuroprotective role (Meltser et al., 2008).

In human studies, ERα have only been found in the spiral ganglion, and ERβ only in stria vascularis cells, which are essential to signal transmission and cochlear homeostasis, respectively, (Stenberg et al., 2001). The presence of ERα and ERβ in the ear affect auditory function in humans in a number of ways. First, estradiol receptors mediate the role of estradiol on the neuronal plasticity, and the metabolic levels of neurotransmitters and blood flow (Stenberg et al., 1999; Caruso et al., 2000; Lee and Marcus, 2001). Second, while ERα and ERβ are found in both men and women, their expression is related to the level of estradiol in the blood serum (Hultcrantz et al., 2006; Motohashi et al., 2010), and this level fluctuates over time in women. Additionally, the up- and down-regulation of ERα and ERβ in the inner ear depends on the life stage of a women (Simonoska et al., 2009), such that the level of estradiol influences auditory function in different ways at different times, in particular during maturation of the organism, the menstrual cycle, pregnancy, and menopause (Al-Mana et al., 2008; He and Ren, 2018).

In animals, ERα and ERβ have been found in the inner ear plasma membrane cells, the cochlear and vestibular fluids, cochlear cells including the OHCs, inner hair cells (IHCs), stria vascularis, spiral ligament, Reissner’s membrane, and spiral ganglion cells (Stenberg et al., 1999), and distributed throughout the whole auditory pathway (Stenberg et al., 1999; Charitidi et al., 2009, 2010; Charitidi and Canlon, 2010). Estradiol receptors have also been found in the central nervous system (Contoreggi et al., 2021). In mice, ERα and ERβ were found in the ventral cochlear nucleus, nucleus of the trapezoid body, the lateral- and medio-ventral periolivary nuclei, the dorsal lateral lemniscus, and the inferior colliculus. In lateral olive, the ventral lateral lemniscus and central nucleus of the inferior colliculus only ERβ were found and in the auditory cortex only ERα were found (Charitidi et al., 2010). Similar to human studies, animals showed better hearing sensitivity during higher levels of estradiol (e.g., Sisneros et al., 2004; Arch and Narins, 2009; Frisina, 2012). No sex differences have been found in the expression patterns of estradiol receptors in the central auditory system neither in young nor aged mice (Charitidi and Canlon, 2010; Charitidi et al., 2010).

Regarding potential underlying mechanisms, estradiol has been suggested to play a role in aiding neural excitation in the inner ear and increase the neurosteroids in the brainstem, enhancing the transmission of the auditory signals to the brain (Tremere et al., 2011).

#### 1.3.2. Progesterone receptors

In contrast to estradiol, there is no evidence of the presence of progesterone receptors in the inner ear in either humans or rats (Bonnard et al., 2013). No staining of progesterone receptors observed in stria vascularis, the organ of Corti or the spiral ganglion in either human or rat inner ears. However, progesterone receptor-B was found in the cochlear bone (Bonnard et al., 2013).

### 1.4. Functional evidence for the influence of sex hormones on hearing

The human auditory system shows a number of minor but significant functional sex differences (McFadden, 1998). These differences can be found in both the peripheral and central auditory pathways.

#### 1.4.1. Peripheral auditory function

In terms of overall sex differences in the cochlear function, women have been shown to have better (more sensitive hearing) PTA thresholds than men across all frequencies (0.25–8 kHz) (Grinn et al., 2017). OAEs also show significant sex differences both related to their presence and strength (dB SPL) (McFadden, 1998). Specifically, women’s cochleas are more likely to produce SOAEs than men’s. The prevalence varies between studies, 70 vs. 60% (Penner and Zhang, 1997) or 85 vs. 45% (Talmadge et al., 1993), but the overall picture is similar. Snihur and Hampson (2011) found no sex effect in prevalence of SOAEs, but in the strength of SOAEs, with women having significantly stronger SOAEs than men. Burns et al. (1992) found significant sex differences in SOAE prevalence, not only in adults but also in neonates, with females having a higher number of SOAEs present than males. A potential explanation for these findings might be that in female neonates, umbilical cord blood at birth has higher levels of estradiol (Kuijper et al., 2013) than in male neonates. However, by the age of 24 months, these sex differences in SOAEs disappear, possibly because of decreased sex hormones levels in blood and the changes in the external and middle ears (Folsom et al., 1994). In terms of TEOAEs, sex differences have been shown for women (Burns et al., 1992; Shuster et al., 2019) and neonates (Burns et al., 1992; Newmark et al., 1997) with females having stronger TEOAEs than males, but not for older infants (Folsom et al., 1994; Newmark et al., 1997). Newmark et al. (1997) also found that there were fewer asymmetries recorded between both ears in women compared to men.

In contrast to SOAEs and TEOAEs, DPOAEs show no effect of sex hormones. The sex differences found in DPOAEs’ phase delay (longer for men than women) can be fully explained by sex differences in the anatomical length of the cochlea rather than the differences in sex hormones (Bowman et al., 2000).

Sex differences in cochlear function may also contribute to differences in susceptibility to haring loss and in particular noise induces hearing loss. Estradiol can have protective role in the inner ear against noise exposure. Sex differences have been found in prevalence of noise-induced hearing loss (NIHL) between women and men (Pearson et al., 1995; Delhez et al., 2020; Wang et al., 2021). For instance, Wang et al. (2021) conducted a cross sectional study to investigate sex differences in NIHL among 2,280 industrial noise-exposed shipyard workers (1,140 women) and found that women were less likely to develop high-frequency hearing loss than men. It is important to note though that studying the effect of sex hormones on NIHL is particularly challenging in humans, as men are more likely to be exposed to excessive occupational noise than women. Therefore, matching the amount of noise exposure in the participants in order to give a clear view on the protective role of female hormones can be difficult.

Given the absence of progesterone receptors in the cochlea it is unlikely that progesterone has a direct effect on peripheral hearing in humans (Bonnard et al., 2013). However, progesterone receptors have been suggested to play an important role in the central auditory system by modulating the processing of auditory clues (Mann et al., 2012; Upadhayay et al., 2014).

Besides overall static differences in peripheral hearing function due to sex hormones, and particularly estradiol, dynamic sex differences have also been found during the ovarian cycle. Higher levels of estradiol (during the late follicular phase) have been suggested to be associated with a positive effect on hearing sensitivity as evaluated by audiometric threshold (Al-Mana et al., 2010). In particular, PTA thresholds have been reported to improve during higher levels of estradiol (Souza et al., 2017; Emami et al., 2018; Karaer and Gorkem, 2020). In addition, high levels of female hormones during the menstrual cycle have been found to increase the right ear advantage in women (Cowell et al., 2011; Carneiro et al., 2019) with significant differences being reported for cycle phases with high estradiol levels, i.e., follicular phase (Cowell et al., 2011).

As in human studies, animal studies have suggested a positive relationship between levels of estradiol and hearing sensitivity. During high levels of estradiol, better hearing responses were reported in female mice (e.g., Laugel et al., 1987; Canlon and Frisina, 2009; Frisina, 2012), fish (Sisneros et al., 2003, 2004), and frogs (Arch and Narins, 2009).

In addition, estradiol replacement therapy in ovariectomized rats results in a significant improvement in blood circulation in the cochlea (Laugel et al., 1987; Stenberg et al., 2003). This occurs possibly because estradiol inhibits ion transport from stria vascularis by enabling the ion channels in the stria vascularis to inactively secrete K^+^ into the scala media, which in turn enhances the function of OHCs and IHCs (Lee and Marcus, 2001).

The pattern of systematic sex differences in SOAEs and TEOAEs but not DPOAEs has also been found in Rhesus monkeys (McFadden et al., 2005, 2006). McFadden et al. (2006) recorded OAEs in Rhesus monkeys prior to, during and post-breeding season. Female Rhesus monkeys showed stronger and more numerous SOAEs and TEOAEs than male Rhesus monkeys, with female TEOAEs being particularly high during the breeding season (higher estradiol and progesterone levels). No significant sex differences were found in their DPOAEs. There were also no differences in the DPOAEs during the breeding season when the differences in TEOAEs were highest (McFadden et al., 2006). As was already indicated by human studies DPOAEs do not appear to be sensitive to detecting sex differences or changes in cochlear function due to differences in hormone levels.

In studying the effect of progesterone on hearing, Price et al. (2009) found a significant reduction in hearing sensitivity in mid and high frequencies (in ABR and DPOAEs results) in ovariectomized mice that were treated with estradiol-progesterone hormone replacement therapy (HRT). The group of mice that were treated with estradiol monotherapy showed better results compared to the group that was treated with progesterone-containing HRT (Price et al., 2009).

Milon et al. (2018) studied the protective role of estradiol and sex differences in susceptibility to noise exposure in mice. They explosed male and female mice to 2 h of an octave-band of noise centered at 11.3 kHz (8–16 kHz), presented at 101 dB SPL, and found that female mice had significantly smaller permanent threshold shift at 16, 24, and 32 kHz than male mice. This result is in agreement with Meltser et al. (2008) who found that young female mice were more protected from acoustic trauma (12–25 dB threshold shift) than young males (15–26 dB threshold shift) and older female mice (32–49 dB threshold shift) across tested frequencies from 8 to 20 kHz.

#### 1.4.2. Central auditory function

One measure of central auditory function, ABR, shows sex differences in its latencies and amplitudes of response. Specifically, pre-menopausal women have been shown to have larger amplitudes and shorter latencies ABRs (better ABRs) than age-matched men (Zakaria et al., 2019). McFadden (1998) and Meltser et al. (2008) showed that when levels of estradiol concentration in the inner ear were high, wave I latency of ABR decreased (indicating faster conduction) and the ABR amplitude increased, presumably because estradiol improves the neurotransmission of the acoustic signals. Systematic sex differences have been shown also for wave V of ABR, with shorter latencies and larger amplitudes in women than man. While the majority of these differences are thought to be due to differences in head size rather than hormones (women tend to have smaller heads compared to men resulting in a faster propagation of wave V and thus shorter latencies (Don et al., 1993). However, this anatomical difference cannot explain all differences between women and men. Don et al. (1993) showed that the sex differences remained in ABRs even when the size of participants’ heads was considered, suggesting that there might be some role for hormones after all. This interpretation is supported by findings from Zakaria et al. (2019), who recorded ABRs at supra-threshold and threshold levels in young adults from both sexes, while considering comparative head size. They found consistent sex differences in the ABRs with women having better responses (shorter latencies and higher amplitudes of ABRs) at the supra-threshold levels than men (Zakaria et al., 2019).

The relationship between sex differences and speech perception is rarely mentioned in the literature and information on the differences between sexes in speech perception is limited. Wadnerkar et al. (2008), using consonant-vowel (CV) syllable perception to study sex differences in dichotic listening, reported sex differences in dichotic listening asymmetry at lower estradiol levels, but not at higher estradiol levels. Using dichotic digits, staggered spondaic word, and dichotic consonant-vowel tests to study dichotic listening during the menstrual cycle, Carneiro et al. (2019) found sex differences during periods of high levels of estradiol in women when compared to a control group of men. Specifically they found that the right ear in women (compared with the left ear and test session in men) significantly differs during periods of high levels of estradiol in staggered spondaic word and dichotic consonant-vowel tests, but not in dichotic digits (Carneiro et al., 2019).

Sex differences in overall hearing function are further complicated by short-term fluctuations in sex hormones that occur during the ovarian cycle. Dehan and Jerger (1990) reported changes in ABRs that occurred in synchrony with monthly changes in female sex hormones, indicating a possible influence of cyclical sex hormone fluctuations on latencies of ABRs. The nature of this influence is still unclear, with some recent studies suggesting that the effect of estradiol on ABRs may be negative, such that high levels of estradiol prolong latencies of ABRs (Disney and Calford, 2001; Al-Mana et al., 2010).

Fewer studies on the cyclical effect of hormones on hearing in animals are available. Sisneros et al. (2003) investigated cyclical changes in hearing and found that during breeding seasons (higher estradiol levels) the auditory nerve of female Midshipman fish (who have a vocal form of breeding) showed an increase in response to male mating fish. Moreover, when female midshipman fish were treated with estradiol during non-breeding seasons (lower estradiol levels), it resulted in an increase in the sensitivity of their auditory nerve (Sisneros et al., 2004).

The only one study suggesting that progesterone receptors may play an important role in the central auditory processing, and specifically in modulating the processing of auditory clues, comes from túngara frogs (O’Connell et al., 2011). O’Connell et al. (2011) measured auditory activities in anterior, lateral, and ventral thalamic nuclei, as these regions contain progesterone receptors. It was found that progesterone may act as a processing modulator of the auditory inputs. In addition, progesterone receptors were found in both the striatum and medial pallium in this species, which provides another path of progesterone modulation of the auditory input.

### 1.5. The effect of reduced levels of estradiol on auditory function

In both animal and human studies, estradiol has been reported to have multiple protective properties in the inner ear (Mitre et al., 2006; Tremere et al., 2009) and to contribute to protecting the ear from noise exposure, to delay the onset of age-related hearing loss, and to aid spontaneous recovery from sensory-neural hearing loss (e.g., Köşüş et al., 2012; Zhang et al., 2018; Delhez et al., 2020). Therefore, reduced levels of estradiol may cause hearing loss, in particular in menopause, and in Turner Syndrome.

#### 1.5.1. Menopause

Sex differences related to menopause have been reported in terms of onset and severity of age-related hearing loss (ARHL). While men develop ARHL before age-matched women (Davis, 1995), women experience a faster decline in hearing than men after menopause (Hederstierna et al., 2010; Villavisanis et al., 2018). Indeed, the earlier reported advantage in hearing sensitivity for pre-menopausal women compared to men reverses with age (McFadden, 1993) with older women having worse thresholds (i.e., worse hearing sensitivity) than age-matched men (Corso, 1968; Mościcki et al., 1985; Jerger et al., 1993; Kim et al., 2010).

While it has been suggested that hormonal changes in menopause may cause ARHL in post-menopausal women (Wharton and Church, 1991), the actual mechanism of the effect of the reduced levels of hormones on hearing sensitivity for this age group is unclear. Evidence that lower levels of estradiol may play a critical role comes from Karaer and Gorkem (2020) who reported no differences in hearing between pre-menstrual women with premature ovarian failure and post-menopausal women. Similarly, Kim et al. (2002), who studied the association of serum estradiol levels and hearing sensitivity in post-menopausal women, found that lower levels of estradiol increased the risk of hearing loss. In addition, Arora et al. (2021) compared the ABRs of post-menopausal women and pre-menopausal women. They reported that post-menopausal women had significantly reduced amplitudes and prolonged latencies of ABRs. On the other hand, non-significant differences in ABRs using sensation level as stimulus between older men compared to young men found by Anias et al. (2004). Rosenhamer et al. (1980) found non-significant differences in ABRs between post-menopausal women compared to age-matched men and young men. This may concluded that sex hormones may influence ABRs.

Recent attention has focused on the effect of hormone replacement therapy for improving hearing ability in post-menopausal women. According to studies that examined the connection between female sex hormones and hearing, hormonal treatments tend to delay hearing loss in post-menopausal women (Kilicdag et al., 2004; Köşüş et al., 2012; Lee et al., 2017). Furthermore, post-menopausal women who undertook hormonal therapy had better hearing sensitivity than women who did not take HRT. Kilicdag et al. (2004) studied two groups of postmenopausal women, where only one group was given estrogen treatment. They reported that hearing sensitivity at 250–2,000 Hz was better in the group who had the estrogen treatment compared to the control group. In addition, Caruso et al. (2003), when investigating auditory function of women with induced early menopause due to medical intervention, found a decline in auditory function. However, with low doses of estrogen treatment, hearing function improved as demonstrated by shortened latencies of ABRs (Caruso et al., 2003). Even though estrogen hormonal treatment could be a novel approach to restoring and delaying hearing loss, there is a controversy regarding its potential for increasing the risk of developing breast cancer. A randomized placebo-controlled study, however, showed that only a estrogen and progestin combined HRT increased the risk of breast cancer, while an estrogen-only HRT significantly decreased the risks (Chlebowski et al., 2020). In addition, a recent review found that estrogen HRT not only has the potential to prevent breast cancer, but may also be able to help prevent other disorders (e.g., osteoporosis and cardiovascular disease) (Manyonda et al., 2022).

#### 1.5.2. Turner syndrome

Turner syndrome represents another example of the consequences of lack of estradiol for hearing. Turner syndrome is a genetic condition in women caused by either complete or partial deletion of the X chromosome that leads to ovarian dysgenesis and little or no estradiol production. Turner syndrome has been associated with low level of estradiol, which in turn has been suggested to play a critical role in development of hearing impairment (Morimoto et al., 2006; Hederstierna et al., 2009). As secretion of female sex hormones only starts to increase in girls with puberty, and lack of secretion and resulting hearing loss is normally only detected after girls start puberty. Stenberg et al. (1998) reported that young girls with Turner syndrome had within-normal hearing levels before puberty (i.e., as the peak of the sensorineural dip did not exceed 20 dB HL), however, their hearing sensitivity to high frequencies decreased after puberty (i.e., the dip between 25 and 35 dB HL).

#### 1.5.3. Pregnancy

As mentioned earlier, changes in the circulating levels of female sex hormones may affect the functioning of the auditory system. This is also seen during pregnancy (Sennaroglu and Belgin, 2001). Progesterone is considered the main sex hormone during pregnancy, as it is essential in fetus implementation and pregnancy maintenance (Di Renzo et al., 2005). The production levels of progesterone increase significantly during pregnancy (from 0.1 to 40 mg/24 h in non-pregnant women to 250–600 mg/24 h in near-term pregnant women; Sennaroglu and Belgin, 2001).

There is some evidence that low-frequency hearing is slightly elevated in pregnant women, particularly in the third trimester (Sennaroglu and Belgin, 2001). This is most likely due to fluid retention in the inner ear. However, this elevation tends to remain within normal clinical levels. In addition to changes in hearing sensitivity, reduction in DPOAE have been found with DPOAE being absent in 26% of the pregnant as opposed to 4% of the non-pregnant women (Ashok Murthy and Krishna, 2013).

A possible explanation for these changes in hearing function is the substantial increase in progesterone levels in pregnancy, which can lead to edema. Edema can have an effect similar to endolymphatic hydrops in the cochlear aqueduct and essentially lead to a temporary conductive loss. During the post-partum period, when progesterone is reduced again, hearing levels have been found to spontaneously recover (Sennaroglu and Belgin, 2001; Kenny et al., 2011).

### 1.6. Contribution to the field

Sex affects hearing function, yet its effect is regularly ignored. This changed only in 2016 when in the UK sex was added as a biological variable in preclinical research by the National Institutes of Health (Clayton and Collins, 2014); in earlier studies, sex was commonly not reported and analyzed separately, opening the door to the possibility that existing sex differences in the data were not discovered and may have inadvertently affected the results. Based on the results reviewed so far, this bias is most likely to have affected studies of ARHL and NIHL.

Besides a general lack of focus on sex differences in hearing research, there is also the problem of comparability of methodologies for measuring hearing function and hormone levels. One case in point is measures used assess the point of the menstrual cycle. While the most accurate method would be to measure hormone levels in the blood of the participant at the point of auditory testing, most previous work has used self-reported measures. However, we know that self-report measures are less accurate that biological assessment at estimating levels of estradiol in the bloodstream (Farrar et al., 2015), yet studies using either method are treated as comparable. This can make is difficult to develop a clear understanding of how sex hormones affect the various stages of the auditory pathway in an overall and cyclical fashion.

A lack of accuracy in measures and consistency and reliability between measures makes it difficult to combine data from existing studies into in a meta-analysis to obtain a clearer picture of the effects of sex hormones on hearing. As a result, there has been no systematic review of the differences in the auditory function between women and men, and the effect of female hormones fluctuations on auditory function across a specific period (i.e., during menstrual cycle and after menopause) to date. In addition, the possible effect of the female hormones on auditory dysfunction, such as perception of tinnitus and vestibular dysfunction, is unclear. Therefore, this review aims to systematically assessed the literature to give an overview of the methodologies used in research and to identify the gap in knowledge and to make some recommendations for future work to have a better understanding of the association between the levels of these hormones and hearing. This will provide information about how to manage hearing loss, tinnitus, and vertigo in women.

This systematic review aims to answer the following questions:

Review question 1. Does auditory function differ between women and men across the entire lifespan or during part of it?

Review question 2. Does auditory function in women fluctuate over the course of the menstrual cycle?

Review question 3. Does this fluctuation co-vary with changes in female hormone levels?

## 2. Review methods

The protocol of this review was registered with the International Prospective Register of Systematic Reviews (PROSPERO; Reference ID: CRD42020201480) in October 2020 (NIHR, 2020).

https://www.crd.york.ac.uk/prospero/display_record.php?ID~=~CRD42020201480. PRISMA (Preferred Reporting Items for Systematic Reviews and Meta-Analyses) guidelines were used to formulate the eligibility criteria (Moher et al., 2015; Page et al., 2021).

### 2.1. Eligibility criteria

#### 2.1.1. Participants

• Studies of pre-menopausal women/and age-matched men with normal hearing.
• Studies of pre-menopausal women with a regular menstrual cycle, no use of hormonal contraceptives, no pregnancy, and no lactation.
• Studies of post-menopausal women/and age-matched men with normal hearing/hearing loss.

#### 2.1.2. Intervention/Exposure

Estradiol and progesterone.

#### 2.1.3. Comparators

If reported, age-matched men.

#### 2.1.4. Outcomes

Measures of peripheral and central auditory function. Peripheral auditory function: pure- tune audiometry (PTA, conventional and extended high frequencies), tympanometry, medial olivocochlear reflex (MOC) and otoacoustic emissions (OAEs); central auditory function: auditory brainstem responses (ABR), auditory steady state responses (ASSR), speech audiometry, auditory evoked/event-related potentials (AEP and ERP) and any further recommended procedure (e.g., dichotic listening).

#### 2.1.5. Study designs

Randomized controlled trials, non-randomized controlled trials, case- control, cross-sectional and/or prospective cohort/longitudinal studies.

### 2.2. Information sources

The following electronic databases were searched (EMBASE, PubMed, MEDLINE (Ovid), PsycINFO, ComDisDome, CINAHL, Web of Science, and CENTRAL via Cochrane Library). Additional to the electronic databases, reference lists of relevant studies and reviews were scanned, and relevant internet resources (e.g., Mendeley) were searched for relevant publications published between 1999 and 2022. The search strategy is in the Supplementary material.

#### 2.2.1. Selection process

The title and abstract were screened for all retrieved articles. The eligibility of the retrieved articles was assessed according to the inclusion/exclusion criteria by NA. In case of uncertainty, this was solved by discussion with AH and KK.

##### 2.2.1.1. Inclusion criteria

• Published studies in English, or if English translation was available.
• Studies done on human participants, adults (≥17 years).
• Only human participants were included:
  ∘ Pre-menopausal women.
  ∘ Post-menopausal women.
  ∘ Adult men.

##### 2.2.1.2. Exclusion criteria

• Gray literature, systematic review, conference abstracts, book chapters, dissertations, theses, and clinical guidelines.
• Pre-clinical studies/Animal studies.
• Studies that included female participants who were breastfeeding, pregnant or the use contraceptive pills or if not mentioned.
• Studies including participants with additional health conditions or risk factors for ototoxicity, noise exposure and middle ear pathologies.

### 2.3. Data management

The identified papers were extracted to EndNote X9 (Clarivate Analytics, 2018) for the initial screen. Duplicates were removed prior to the screen using the same software. The reviewer NA transferred the following information into an Excel spreadsheet: the titles, authors’ names, year of publication, settings, participants characteristics, publication journals, study design, abstracts, number of sessions, outcome measures (including hormones levels measures), and findings. The excluded papers were documented in the spreadsheet with the reason for exclusion.

### 2.4. Risk of bias assessment

The risk of bias of each individual study was assessed using Newcastle-Ottawa Scale (NOS). This scale judges the quality of papers in three broad perspectives: the selection, comparability, and outcome. In addition, NOS assess the following: control cohort, the number of session (the length/follow up), and outcomes measures (objective or self-reported). The quality of the studies could be judged as either good (low risk), fair (high risk), or poor (very high risk) by awarding stars in each domain accordingly with NOS guidelines (Wells et al., 2000).

### 2.5. Data analysis

The information collected from the systematic review was analyzed qualitatively and represented in tables and paragraphs form. Such material would include participant characteristics, test criteria, outcome measures, and findings. This review did not use meta-analysis due to the amount of the missing data (i.e., SD, and number of participants in each group were not reported). Attempts were made to contact the author(s), but this information was not provided. Therefore, each paper was assessed to reach a general conclusion.

### 2.6. Search results

**FIGURE 2:**
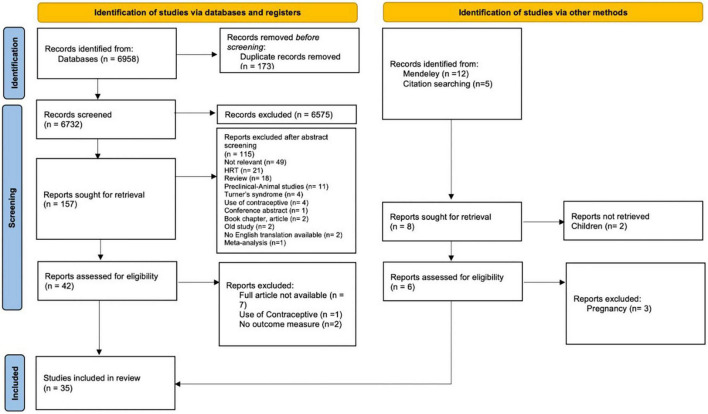
Preferred reporting items for systematic reviews and meta-analyses 2020 selection process flow.

The initial search of the databases recorded 6,958 articles. 173 articles were duplicates and removed by automation tools. After removal of duplicates, titles were screened to identify relevant studies. The screening identified 6,732 potential articles. An additional 17 articles identified through Mendeley, and hand search (i.e., checking references and citation). After screening the titles, 165 articles remained for abstract screening. After abstract screening, 119 articles were excluded. The full text assessment of the remained 48 articles resulted in identifying 35 articles that meet the inclusion criteria. A summary of the selection process is presented in the PRISMA flow chart diagram (Figure 2).

### 2.7. Study characteristics

The included studies were divided into three groups based on participant characteristics and study design: studies on the sex differences between pre-menopausal women and age-matched men in auditory function, studies on the female hormones’ fluctuation in pre-menopausal women, and auditory changes in post-menopausal women.

Changes in levels of female sex hormones were measured using biological samples (i.e., blood or saliva, used in 11 studies) or self-reported measures (i.e., day counting, used in 7 studies) to predict the phase of the menstrual cycle and then infer the level of female sex hormones. This was done by counting the day of the cycle according to the participants’ average menstrual cycle length (i.e., this is calculated from the first day of last menstrual period). Six studies were unclear on the methods used to measure the female hormones.

#### 2.7.1. Sex differences between pre-menopausal women and age-matched men

Eleven studies investigated sex differences in the peripheral and central auditory system (Bowman et al., 2000; Ismail and Thornton, 2003; Dreisbach et al., 2007; Sharashenidze et al., 2008; Kim et al., 2010; Snihur and Hampson, 2011; Jalaei et al., 2017; Boothalingam et al., 2018; Melynyte et al., 2018; Stuart and Kerls, 2018; Zakaria et al., 2019). A summary of the characteristics of the studies are presented in Table 1.

**TABLE 1:** Summary of the characteristics and results of studies on “sex differences in auditory function.”

References	Study design	Sample size	Age mean	Hearing level	Focus	Outcome measures	Findings
Bowman et al., 2000	Comparative study	Women (30) Men (30)	19-35 years women (25.0 years) men (25.6 years)	Normal hearing	Sex differences in DPOAE’s	DPOAE recordings	There are sex differences in DPOAEs recordings, but these differences are related to the anatomical differences in cochlear length between sexes, not differences in hearing sensitivity. At low frequencies, men had longer DPOAE measures than women.
Ismail and Thornton, 2003	Comparative Study	Women (40) ears Men (41) ears	18-40 years	Normal hearing	Sex differences in MLS OAE	MLS OAEs recordings	There are sex differences in MLS OAEs. Women had a greater MLS OAEs amplitude than men. The relevance of this difference, however, diminishes as the click stimulation rate increases. Women’s right ears reported to have greater MLS OAEs amplitude of than women’s left ears.
Dreisbach et al., 2007	Comparative Study	Women (30) Men (30)	18-39 years	Normal hearing	Race and sex differences in PTA and DPOAEs	PTA DPOAEs	Women had better hearing sensitivity at 14 K and 16 KHz. No racial or sex differences were found for the DPOAE measure.
Sharashenidze et al., 2008	Comparative study	Women (128) Men (96)	30-79 years	Hearing levels varied among the age groups and sex	Sex differences in age-related hearing loss/presbycusis	PTA	Women had better hearing levels than men in age groups of 30-39, 40-49 and 50-59 years. In the age group of 60-69 and 70-79 years, women tend to have a steeper decrease in hearing, and the sex differences in hearing sensitivity are smoothed significantly.
Kim et al., 2010	Comparative study	Women (902) Men (214)	15-83 years women (46 years) men (47.6 years)	Young group: normal hearing Old group: ARHL	Sex differences in ARHL	PTA	There are significant sex differences in PTA thresholds. Women have better hearing at higher frequencies than men. At 4 kHz and 8 kHz, men reported to have greater age-related changes in hearing than women.
Snihur and Hampson, 2011	Comparative study	Women (48) Men (45)	17-25 years women (19.9 years) men (20.8 years)	Normal hearing	Sex differences in SOAE and CEOAE	SOAE CEOAE recordings	There are sex differences in SOAEs and CEOAE. Women producing more numerous and stronger SOAEs, and CEOAEs with greater response amplitude compared to men.
Jalaei et al., 2017	Comparative study	Women (15) Men (14)	19-30 years Women (23.5 years) Men (22.7 years)	Normal hearing	Sex differences in speech-ABR	Speech-ABR	Significant sex differences in the amplitude of speech-ABR peaks V and A. Higher amplitudes and less steep V/A slopes were observed in women than in men, and these differences persisted when considering differences in head size. Women were found to have shorter latencies of peak V and A. However, the differences in latencies were insignificant when considering the differences in head size.
Boothalingam et al., 2018	Comparative study	Women (522) Men (365)	10-68 years	Normal hearing	Sex, race, ear differences in DPOAE’s	DPOAE recordings	There are no significant sex differences in DPOAE recordings found in the study.
Melynyte et al., 2018	Comparative study	Women (22, 11 left-handed) Men (22, 11 left-handed)	left-handed (23 years) right-handed (22 years)	Normal hearing	Sex differences and handedness in 40 Hz ASSR	40 Hz ASSR	There are sex differences observed in the left-handed participants, as women significantly had lower phase-locking and event-related spectral perturbation values of 40 Hz ASSRs compared to the left- handed men. However, no significant sex differences between right-handed women and men.
Stuart and Kerls, 2018	Comparative study	Women (50) Men (50)	Women (22.1 years) Men (23.4 years)	Normal hearing	Sex differences in contralateral inhibition of transient evoked otoacoustic emissions (TEOAEs)	Contralateral TEOAEs	There are significant sex differences in TEOAEs recording. The levels of TEOAEs were larger in women and in the right ear than in men and the left ear. There is no significant effect of ear or sex on absolute TEOAEs inhibition. Significant negative correlations and linear predictive relations were found between TEOAE levels and normalized TEOAE inhibitions in both ears. There is no evidence of the same with absolute inhibition of TEOAEs. The effect of ear and sex on normalized inhibition are small and may have no clinical or practical significance.
Zakaria et al., 2019	Comparative study	Women (17) Men (13)	Women (22.6 years) Men (21.9 years)	Normal hearing	Sex differences in ABR at Suprathreshold	ABR	A significant sex differences in ABR results among young adults were found at suprathreshold levels. These differences are not related to the head size. Normative data for sex differences in ABR are valuable for clinical applications, particularly at high stimulation levels.

The sample size ranged from 29 to 1,116 participants. Eight studies had similar participant characteristics; young adults with normal hearing (Bowman et al., 2000; Ismail and Thornton, 2003; Dreisbach et al., 2007; Snihur and Hampson, 2011; Jalaei et al., 2017; Melynyte et al., 2018; Stuart and Kerls, 2018; Zakaria et al., 2019). Three studies included young and older participants. In the Boothalingam et al. (2018) all participants had normal hearing; however, in Sharashenidze et al. (2008) and Kim et al. (2010), older participants reported to have ARHL. In addition, for older participants no history of excessive noise exposure was reported in these studies. Six studies reported OAE outcomes, three PTA, 2 ABR, and one ASSR outcomes. Most outcomes were reported for young adults (eight studies), but three studies reported outcomes across the whole adult age range. In all cases the studies only comprised one testing session.

#### 2.7.2. Female hormone fluctuations in pre-menopausal women

Nineteen articles studied the effect of female sex hormone fluctuations on auditory function throughout the auditory pathway in women across the menstrual cycle (Serra et al., 2003; Yadav et al., 2003; Walpurger et al., 2004; Wadnerkar et al., 2008; Al-Mana et al., 2010; Cowell et al., 2011; Hjelmervik et al., 2012; Mann et al., 2012; Griskova-Bulanova et al., 2014; Upadhayay et al., 2014; Hodgetts et al., 2015; Adriztina et al., 2016; Batta et al., 2017; Hu and Lau, 2017; Liu et al., 2017; Souza et al., 2017; Emami et al., 2018; Carneiro et al., 2019; Karaer and Gorkem, 2020). A summary is presented in Table 2.

**TABLE 2:** Summary of the characteristics and results of studies on “the fluctuation of auditory function during the menstrual cycle.”

References	Study design	Sample size	Age mean (SD)	Hearing level	Control group	Number of sessions	Experimental group description	Outcome measures		Findings
								**auditory**	**hormones**	
Serra et al., 2003	Observational study	Women (94)	27.9 (6.1)	Normal hearing	No control	Three sessions Early follicular phase (day 5-8) Late follicular phase (day 13-16) Early luteal phase (day 18-23)	Regular cycle (28.3, SD 3.3)	ABR	Enzyme-linked immunosorbent assay	Shorter wave latencies and interpeak intervals during the late follicular phase than during the early luteal phase.
Yadav et al., 2003	Observational study	Women (40) [20 women use contraceptive pills (CP)]	19-26	Normal hearing	age-matched women taking hormonal contraceptive pills	Four sessions in a single cycle 1. Early follicular phase (day 1-3) 2. Late follicular phase (day 11-14) 3. Early luteal phase (day 17-22) Late luteal phase (day 25-27)	Regular menstrual cycles Anovulatory cycle/use of contraceptive pills	LLAEPs	Day counting	P2 and N2 latencies varied significantly throughout the phases of the cycle in normal cycling women. The latencies increased from early to late follicular phase and decreased during early luteal phase and increased again in late luteal phase. Similar but insignificant changes in P1 and N1 were observed. No changes or variation were noticed in CP group, LLAEPs remained consistent.
Walpurger et al., 2004	Observational study	Women (18)	18-35 years 26.5 (5.7)	Normal hearing	No control	Three sessions 1. Early follicular phase 2. Late follicular phase 3. late luteal phase	Regular cycle (24-35 days) No use of contraceptive pills	Event-related potentials (ERPs)	Saliva sample	There are changes in auditory ERPs across the menstrual cycle. The most prominent changes were observed during the late luteal phase, where the vertex potential was significantly reduced compared to menses and to the follicular phase. Which suggests that during high estradiol and progesterone levels in the luteal phase, the involuntary cortical arousal response to the external stimuli is reduced.
Wadnerkar et al., 2008	Observational study	Women (25) Men (20)	Women: 22.56 (2.04), Men: 22.15 (1.69)	Normal hearing	Age-matched men	Women tested in two sessions during one cycle: Early follicular phase (day 2-5) Between two phases, the early and late luteal phase (day 18-25) Men tested once	Regular menstrual cycle (29.24 days, SD2.45),	Dichotic CV stimuli	Day counting	No significant effect of the menstrual cycle on dichotic listening. Number of responses did not differ between the groups.
Al-Mana et al., 2010	Observational study - longitudinal	Women (16)	31.4 (8)	Normal hearing	No control	Four sessions Early follicular phase (5-8 days) Late follicular phase (10-14 days) Early luteal phase (20-23 days) Late luteal phase (25-28 days)	Regular cycle (28.5, SD 1.6)	SOAEs TEOAEs MOC suppression, ABR	Blood samples	During late follicular phase, SOAE amplitudes were significantly greater. The linear regression analysis of all TEOAEs in four sessions showed no correlation with E2. However, In the early and late follicular phase, positive correlation between TEOAEs and E2 was reported, and negative correlation between MOC and E2. The regression analysis of the correlation between TEOAEs and MOC and progesterone level showed no significant findings. ABRs showed a significant change during the ovarian cycle, with an increase in the wave V latency in the late follicular phase and a decrease in the early and late luteal phase.
**Cowell et al., 2011**	**Observational study- cross-sectional**	**Women (21)**	**25.24 (0.74)**	**Normal hearing**	**No control**	**One session** **8 women started in the early follicular phase, 2 in the late follicular phase, and 11 in the early luteal phase.**	**regular cycle (29.20, SD 0.96)**	**CV dichotic tests**	**Blood sample**	**Sex differences in dichotic listening found to be dependent to the hormonal status in women.** **Increases in the right ear advantage (REA) were found in women throughout periods of the menstrual cycle. REA was greater during higher levels of ovarian hormone.** **Left ear scores decreased during higher levels of luteinizing hormones (LH).**
**Hjelmervik et al., 2012**	Randomized Controlled Trial	Women (15) Men (15)	Women 23.5 years (5.1) Men: 23.1 years (2.4)	Normal hearing	Age-matched men	Three sessions for both groups For women: Early follicular phase (day 2-4) Late follicular phase (day 8-12) Early luteal phase (day 20-22)	Regular cycle (26-32 days)	Dichotic testing: CV	Saliva sample	Women perform better in the late follicular phase compared to the early follicular and early luteal phases.
**Mann et al., 2012**	Observational study- cross-sectional	Women (50)	19-36 years	Normal hearing	No control	Four sessions Early follicular phase (day 1-3) Late follicular phase (day 11-14) Early luteal phase (day 17-22) Late luteal phase (day 25-27)	Regular menstrual cycles (28-30 days) and they had not taken any hormonal pills during the past 6 months.	ABR	Day counting	During the late follicular phase, the waves latencies were increased, that showed a slower neural conduction. This can be attributed to the high levels of estradiol during the late follicular phase of the menstrual cycle. The waves latencies decreased in the early luteal phase and hence, this enhanced the conduction across the neural pathways.
**Griskova-Bulanova et al., 2014**	Observational study- cross-sectional	Women (28)	20.68 years (0.63)	Normal hearing	No control	During one of the cycle phases Early follicular phase Late follicular phase Early luteal phase	Regular cycle (28.59, SD 2.13)	40 Hz ASSR	Saliva sample	Significant effect of menstrual cycle phase was seen for the total intensity of 40 Hz ASSRs. ASSR amplitudes were highest during the late follicular phase, intermediate during the early follicular phase and lowest during the early luteal phase. No relationship of any measures to progesterone concentrations was observed.
**Upadhayay et al., 2014**	Observational study- cross-sectional	Women (40)	16-26 years mean: 19 years (2.35)	Normal hearing	No control	Two sessions (one session during the follicular phase and another session during the luteal phase) The testing sessions were reported to be between 2-4 days before ovulation and 9-11 days after ovulation, according to their menstrual cycle. The exact testing days were not reported.	Regular cycle, No use of contraceptive pills, no pregnancy, no lactation	ABR	Day counting	There was a significant variation in ABR waves in the menstrual cycle. Better ABR recordings were observed during luteal phase compared to follicular phase of menstrual cycle. Progesterone is the likely hormone responsible for the increase in the conduction of auditory pathways in women of reproductive age group.
Hodgetts et al., 2015	Randomized Controlled Trial	Women (73)	23 years (4.86)	Normal hearing	No control	One session: The testing day was selected according to the women’s self-reported cycle day (days 1-4, 7-12, 15-23, corresponding to the menstrual, follicular, or luteal phase, respectively)	Regular menstrual cycle (24-35)	Dichotic CV	Saliva sample	High levels of estradiol reported to reduce the stimulus-driven (bottom-up) aspect of lateralization rather than top-down cognitive control.
Adriztina et al., 2016	Observational study- cross-sectional	Women (49)	20-40 years	Normal hearing	No control	Three sessions during one cycle: Early follicular phase (day 3). Late follicular phase: tested with the ovulatory kit, indicating the estradiol at a high level. Early luteal phase: (day 21-22).	Regular menstrual cycle (24-35)	PTA, Tympanometry DPOAEs	Day counting	There was no significant correlation between menstrual and hearing thresholds. It was reported that during late follicular phase, there was a reduction in hearing sensitivity at 4 kHz in the right ear. However, DPOAEs amplitude significantly increased during late follicular phase, compared to early follicular and early luteal phase. This might suggest a positive effect of high levels of estradiol on the cochlear function.
Batta et al., 2017	Observational study- cross-sectional	Women (80)	18-24 years (18.8)	Normal hearing	No control	Three sessions during one cycle Early follicular phase (day 1-3) Late follicular phase (day 10-12) Early luteal phase (day 20-22)	Regular menstrual cycle and no use hormonal contraceptives	ABR	Not mentioned	There is a decrease in waves latencies during late follicular phase. It was reported that estradiol increases transmission in the auditory pathways, and it might be responsible for the shorter latency values of ABR. However, this variation is not statistically significant. There is no effect of female sex hormones on ABR waves amplitudes.
Hu and Lau, 2017	Observational study - longitudinal	Women (20)	21.5 (0.8)	Normal hearing	No control	Four sessions: Early follicular phase (day 3-4). Late follicular phase (day 9-10). Late follicular phase (ovulation) (day 14-15). Early luteal phase (day 21-22).	Regular cycle	ABR CV	Day counting	Peak V latency reported to be significantly lengthened during late follicular phase, but it is not true at peak I and peak III. The interpeak conduction times of inter-peaks I-V and III-V were prolonged at late follicular phase. It was concluded that the central conduction time depends on the phase of the menstrual cycle, which might affect dichotic listening performance.
Liu et al., 2017	Comparative study	Women (17) Men (18)	24-34 years Women (27.29 years) Men (28.17 years)	Normal hearing	Age-matched men	One session, the day of the cycle was not reported. the levels of estradiol and testosterone concentration were measured after the testing session.	Not mentioned	Speech-ABR	Blood samples	Significant effect of sex hormones on speech encoding in the brainstem. Estradiol is observed to affect the amplitude of neurons but has little effect on the conduction velocity of neurons (latency). Estradiol may improve brainstem auditory neuron excitability and phase-locking ability for speech coding.
**Souza et al., 2017**	Comparative study	**Women (20)** **Men (10)**	**18**-**39 years**	**Normal hearing**	**Age-matched men**	**Four sessions over one cycle** **Early follicular phase (day 1**-**7)** **Late follicular phase (day 8**-**13)** **Early luteal phase (day 14**-**22)** **Late luteal phase (day 23**-**28)**	**10 women who have regular menstrual cycle, and 10 women who use hormonal contraceptive.**	**PTA**	**Day counting**	**There is a significant effect of hormonal fluctuations and hearing thresholds across the menstrual cycle.** **The hearing threshold of women who don’t use contraceptive varied significantly through the cycle with mean variation of 4.09 dB HL. Men hearing threshold did not varied between the sessions.** **For women who did not use contraceptives, the lowest threshold was observed in the late follicular phase.**
**Emami et al., 2018**	Case-control study	Women (20)	19-30	Normal hearing	No control	Two sessions: Late follicular phase (day 13). Early luteal phase (day 22).	Regular cycle (28 days)	PTA Tympanometry DPOAEs ABR	Not mentioned	It was reported that there are individual differences in the effect of female sex hormones on hearing. As the auditory function seems to be sensitive in some women to hormonal changes. During the early luteal phase, high level of progesterone caused a reduction in hearing (worse hearing at 250 Hz), increased DPOAEs amplitude, delayed ABR interpeak latencies). Better hearing sensitivity in follicular phase.
**Carneiro et al., 2019**	Cohort, longitudinal, blinded	Women (9) Men (11)	25 (15)	Normal hearing	Age-matched men	Two sessions for both groups For women: Late follicular phase (day 11-13) Late luteal phase (day 23-26)	Regular menstrual cycles (27 to 32 days)	Dichotic testing: SSW, DD, and CV	Blood sample	In late follicular phase, better responses in women and in the right ear. Estradiol improved dichotic listening in women during higher level of E2 in the menstrual cycle. No significant changes in men performances.
**Karaer and Gorkem, 2020**	Case-control, cross-sectional	Women (90; 30 premature ovarian failure, 30 normal, 30 menopausal)	POF: 32.5 (1.06), menopause 54.4 (1.1), Control 28.4 (1.06)	POF, control: normal hearing. older women: normal hearing, mild HL at 6-8 Hz	Normal pre-menopausal women	Each group was tested in one session: For the POF tested after at least 6 months of the diagnosis (the presence of amenorrhea and a serum FSH level greater than 40 mIU/mL on two occasions at least 1 month apart). For the menopausal women (amenorrhea for at least 1 year). For the control group (normal regular menstrual cycle), no mention of the cycle phase or day of the testing.	Hearing function is impaired in women with premature ovarian failure	PTA TEOAEs DPOAEs	Blood sample	In the POF group, there was a significant decline in the 6 kHz DPOAEs compared with the pre-menopausal group. The menopausal group showed worse hearing which reported to be due to aging and reduced level of estradiol. There was a negative effect of reduced estradiol levels on OHC function, in the POF group. Normal estradiol levels may promote healthy OHC function.

The sample size of these studies ranged from 16 to 94 participants. The participant characteristics are similar in seventeen studies: young adult, normal hearing levels, regular menstrual cycle, no pregnancy, no lactation for 6 months prior to testing. One study included women with premature ovarian failure and normal hearing levels (Karaer and Gorkem, 2020). One study did not mention the regularity of the menstrual cycle of female participants (Liu et al., 2017).

Only seven studies had a control group. In addition, the make-up of control groups differed between studies. In most studies (five), the control were age-matched men (Wadnerkar et al., 2008; Hjelmervik et al., 2012; Liu et al., 2017; Souza et al., 2017; Carneiro et al., 2019). Three studies either exclusively or additionally used control groups comprised of women with premature ovarian failure cycle and older women with ARHL (Karaer and Gorkem, 2020) or women who use a method of hormonal contraception as a control (Yadav et al., 2003; Souza et al., 2017).

The number of sessions varied in these studies. Testing sessions varied between four (Yadav et al., 2003; Al-Mana et al., 2010; Mann et al., 2012; Hu and Lau, 2017; Souza et al., 2017), three (Serra et al., 2003; Walpurger et al., 2004; Hjelmervik et al., 2012; Adriztina et al., 2016; Batta et al., 2017), two (Wadnerkar et al., 2008; Upadhayay et al., 2014; Emami et al., 2018; Carneiro et al., 2019), and one (Cowell et al., 2011; Griskova-Bulanova et al., 2014; Hodgetts et al., 2015; Liu et al., 2017; Karaer and Gorkem, 2020). In studies with more than one session, there was at least an element of repeated testing to compare outcome measures within participants across different phases of the menstrual cycle. In studies with only one session, all comparisons between outcome measures across different phases of the menstrual cycle were between-participant.

#### 2.7.3. Auditory changes in post-menopausal women

Five studies investigated auditory changes in post-menopausal women, with the sample size ranging from 22 to 190 participants (Tandon et al., 2001; Hederstierna et al., 2010; Svedbrant et al., 2015; Trott et al., 2019; Arora et al., 2021). One study tested participants three times (i.e., at 2, 7, and 10 years after the start of menopause) (Svedbrant et al., 2015). One study tested participants twice with mean interval of 7.5 years between the two sessions (Hederstierna et al., 2010). Three studies tested participants once (Tandon et al., 2001; Trott et al., 2019; Arora et al., 2021). A summary is presented in Table 3. None of these studies included age-matched men as a control. In Hederstierna et al. (2010) and Svedbrant et al. (2015) participants had a baseline normal hearing level. In addition, the participants in Tandon et al. (2001), Trott et al. (2019), and Arora et al. (2021) studies reported to have normal hearing level.

**TABLE 3:** Summary of the characteristics and results of studies on “the effect of menopause on hearing function.”

References	Study design	Sample size	Mean age (SD)	Hearing level	Control group	Number of sessions	Experimental group description	Outcome measures		Findings
								**auditory**	**hormones**	
Tandon et al., 2001	Observational study- cross-sectional	Women (22)	Post-menopausal between 50 and 70	Normal hearing	No control	One session	Post-menopausal	ABR	Not mentioned	Post-menopausal women had longer conduction time due to hormonal changes resulting from menopause. Significant increase in wave I, III, V latencies and the interpeak latency between I-III, I-V, and III-V in post-menopausal women
Hederstierna et al., 2010	Observational study - longitudinal	Women (104)	51.2 (1.5)	baseline: normal hearing	No control	Tested twice with an average interval of 7.5 years	Post-menopausal	PTA	Not mentioned	It was reported that a rapid decline of hearing levels in healthy women after the start of menopause, which appears to act as a trigger of age-related hearing loss in women. This decline was noticed to start in the left ear.
Svedbrant et al., 2015	Observational study - longitudinal	Women (100)	49.3 (1.6)	Baseline: normal hearing	No control	2, 7, 10 years follow-up	Post-menopausal	PTA	Blood sample	The hearing level declined rapidly in the peri-menopausal group at 1-3 kHz for both ears, and a rapid decline of hearing was seen after menopause. However, no significant correlation between hormonal levels and hearing levels for this age group.
Trott et al., 2019	A prospective, group comparison study	14 Peri-post-menopausal women	54 years	Normal hearing	Pre-menopausal women	One session	Peri-menopausal women (Having irregular cycles between three and 11 months) Post-menopausal women (Having at least 1 year of amenorrhea)	Dichotic Digit (DD) testing Speech in noise (LiSN-S/SPIN-R) ABR- MLR	Not mentioned	Non-significant differences in DD, SPIN-R tests or MLR between groups. Significant differences in LiSN-S between groups, pre- and post-menopausal women have poor SRT. Significant ABR differences, pre- and post-menopausal women have longer wave V latencies with a higher stimulus rate.
Arora et al., 2021	Cross-sectional	Pre-menopausal women (90) Post-menopausal women (100)	18.6 (0.73) 59.8 (5.84)	Both groups have normal hearing	Pre-menopausal women were tested during follicular phase	One session	Post-menopausal women (at least 1 year of amenorrhea)	ABR	Not mentioned	ABR waves latencies increased in post-menopausal women which show subtle degenerative changes in hearing that start appearing in the central auditory pathway after menopause and probably caused by estradiol decline. As estradiol levels influences the sensory transmission in the auditory pathway.

### 2.8. Outcomes measures

#### 2.8.1. Audiometric measures

Sex differences between pre-menopausal women and age-matched men were assessed across the peripheral auditory pathway using PTA (Dreisbach et al., 2007; Sharashenidze et al., 2008; Kim et al., 2010), SOAEs and COAEs (Snihur and Hampson, 2011), maximum length sequence OAEs (MLS OAEs) (Ismail and Thornton, 2003), contralateral TEOAEs (Stuart and Kerls, 2018), and DPOAEs (Bowman et al., 2000; Dreisbach et al., 2007; Boothalingam et al., 2018). In addition central auditory measures assessed ABR (Zakaria et al., 2019), speech-ABR (Jalaei et al., 2017), and 40 Hz ASSR (Melynyte et al., 2018).

Fluctuations in female hormones in pre-menopausal women were assessed using the following peripheral auditory measures: middle ear function (Adriztina et al., 2016; Emami et al., 2018), TOAEs (Al-Mana et al., 2010; Karaer and Gorkem, 2020), DPOAEs (Adriztina et al., 2016; Emami et al., 2018; Karaer and Gorkem, 2020), PTA (Adriztina et al., 2016; Souza et al., 2017; Emami et al., 2018; Karaer and Gorkem, 2020) and medial olivocochlear suppression (Al-Mana et al., 2010). Central auditory function was assessed using dichotic speech audiometry (Wadnerkar et al., 2008; Cowell et al., 2011; Hjelmervik et al., 2012; Hodgetts et al., 2015; Hu and Lau, 2017; Carneiro et al., 2019), ABR (Serra et al., 2003; Al-Mana et al., 2010; Mann et al., 2012; Upadhayay et al., 2014; Batta et al., 2017; Hu and Lau, 2017; Emami et al., 2018), long latency auditory evoked potentials (LLEAPs) (Yadav et al., 2003), 40 Hz auditory steady-state response (ASSR) (Griskova-Bulanova et al., 2014), event-related potentials (ERPs) (Walpurger et al., 2004), and speech-ABR (Liu et al., 2017).

Auditory changes in post-menopausal women were assessed using PTA (Hederstierna et al., 2010; Svedbrant et al., 2015), dichotic digit test, Speech in noise tests, middle latency response (MLR) (Trott et al., 2019) and ABR (Tandon et al., 2001; Trott et al., 2019).

A summary of the hormonal tests for each study that investigated female hormones fluctuation in pre-menopausal women is presented in Table 2. Only one study measured female hormone levels in post-menopausal women, and they used blood samples (Svedbrant et al., 2015). The method of measuring the level of the hormones was not mentioned in Tandon et al. (2001) and Hederstierna et al. (2010).

## 3. Results

### 3.1. Sex differences between pre-menopausal women and age-matched men

#### 3.1.1. Peripheral auditory function

The findings suggested an overall sex difference for measures of peripheral auditory function, particularly PTA. Specifically, with nine studies out of eleven showed a significant sex-specific differences in the audiometric measures in favor of women with PTA thresholds in adults (between 18 and 49 years) being better in women than men, mainly at higher frequencies (Dreisbach et al., 2007; Sharashenidze et al., 2008; Kim et al., 2010).

Although sex differences were also evident in DPOAEs recordings, they have been suggested to be related to the anatomical differences (i.e., due to the differences in the cochlea length) rather than the biological sex differences (Bowman et al., 2000; Dreisbach et al., 2007; Boothalingam et al., 2018). However, all other types of OAEs suggested significant sex differences in the function of the cochlea [i.e., outer hair cells (OHCs)] independent of its length. Women and right ears were reported to have stronger SOAEs and larger TEOAEs, and contralateral TEOAEs amplitudes (Ismail and Thornton, 2003; Snihur and Hampson, 2011; Stuart and Kerls, 2018).

#### 3.1.2. Central auditory system

ABR recording at suprathreshold levels showed sex differences between pre-menopausal women and men with women showing better responses, i.e., shorter latencies and larger amplitudes (Zakaria et al., 2019). In addition, speech-ABRs showed larger amplitudes and shorter latencies of wave V and A in women compared to men (Jalaei et al., 2017). 40-Hz ASSRs were reported to be better in women than men, however, this was only reported for left-handed but not for right-handed participants (Melynyte et al., 2018).

Evoked potential recordings showed sex differences. Sex differences in ABRs were found only at suprathreshold levels, where women had better responses than men (i.e., larger amplitude and shorter latencies). These differences remained even when controlling for the differences in head sizes (Zakaria et al., 2019) and were reported to be not related to the differences in head size of participants. Don et al. (1993) reported the same findings. In addition, speech-ABRs were also found to be better in women with larger amplitude of waves as compared to men. However, the latencies of speech-ABRs were reported to be related to anatomical differences of the head diameter (Jalaei et al., 2017). The ABR recordings of menopausal women showed prolonged conduction time which was reported to be the driven by changes in female sex hormones levels.

### 3.2. Female sex hormone fluctuation in pre-menopausal women

#### 3.2.1. Peripheral auditory function

Hearing sensitivity was reported to be improved during the late follicular phase of the cycle (i.e., mainly during higher levels of estradiol). PTA thresholds were found to be decreased (i.e., better hearing sensitivity) during the late follicular phase (Adriztina et al., 2016; Souza et al., 2017; Emami et al., 2018; Karaer and Gorkem, 2020).

Spontaneous Otoacoustic Emissions, TOAEs and DPOAEs were better during late follicular phase (Al-Mana et al., 2010; Adriztina et al., 2016; Emami et al., 2018; Karaer and Gorkem, 2020). On the other hand, no significant effect of progesterone on OAEs was reported (Al-Mana et al., 2010).

A negative correlation between MOC suppression and estradiol was reported, and no significant effect of progesterone on MOC suppression (Al-Mana et al., 2010).

#### 3.2.2. Central auditory system

Reports regarding the effect of estradiol and progesterone on ABR wave latencies were inconsistent. Some studies reported increased latencies (i.e., longer transmission time) of ABR I-V waves (i.e., worsening) during the late follicular phase and shorter latencies (i.e., improvement) were reported to be during luteal phase (i.e., during higher levels of progesterone) (Al-Mana et al., 2010; Mann et al., 2012; Upadhayay et al., 2014; Hu and Lau, 2017; Emami et al., 2018). Other studies reported decreased ABR latencies (i.e., improvement) during follicular phase (Serra et al., 2003; Batta et al., 2017). There is an increase in the amplitude of speech-ABR waves during late follicular phase, but no changes in the latencies were reported (Liu et al., 2017). 40-Hz ASSR have been reported to improve during higher levels of estradiol in one study (Griskova-Bulanova et al., 2014).

Long latency auditory evoked responses (LLEAPs) recording was found to fluctuate in women with normal ovulatory cycle, however, there was no fluctuation of LLEAPs recording in women with anovulatory cycle (who use hormonal contraceptives) (Yadav et al., 2003). In addition, better ERPs were reported to occur during luteal phase only, i.e., when the level of progesterone increases (Walpurger et al., 2004). The following figure illustrates the fluctuation of the audiological tests results during the menstrual cycle as reported by the included studies.

**FIGURE 3:**
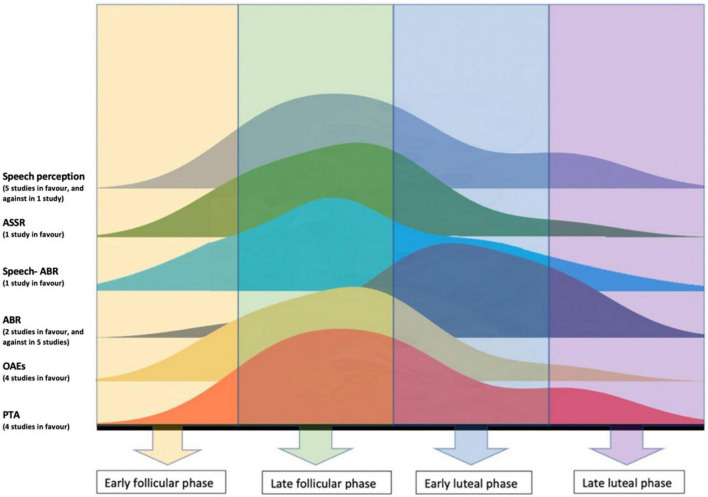
Illustration of the fluctuation of the audiological performances across the menstrual cycle, the peaks represent better performance.

The performance of women in speech audiometry fluctuated through the menstrual cycle. Five out of six studies reported better performance in speech perception during high levels of estradiol (Cowell et al., 2011; Hjelmervik et al., 2012; Hodgetts et al., 2015; Hu and Lau, 2017; Carneiro et al., 2019), whereas the sixth study, (Wadnerkar et al., 2008) reported no significant effect of estradiol in dichotic listening during the follicular phase, and no differences in response number between women and men. Wadnerkar et al. (2008) findings could not reflect the true effect of estradiol in hearing for two reasons. First, the day of the menstrual cycle was self-reported by participants so the level of female sex hormones can only be surmised. Using a self-reported measure to investigate the level of hormones in the body is known not to be accurate. Another explanation to this finding, Wadnerkar et al. (2008) tested women in two sessions: one session in the early follicular phase (day 2–5) which was during low estradiol and progesterone levels, and another session which fell between two phases, the early and late luteal phase (day 18–25). The second session reported by Wadnerkar et al. (2008) to be in the follicular phase and during high levels of estradiol and progesterone. However, since all participants were reported of having normal average menstrual cycle (around 28 days) then this session was undertaken in luteal phase and not the follicular phase. Figure 3 illustrates the fluctuation of the audiological performances across the menstrual cycle phases, where the peaks represent better performance.

### 3.3. Auditory changes in post-menopausal women

A significant rapid reduction in hearing sensitivity after menopause has been reported, particularly at 1 kHz (Hederstierna et al., 2010) and 3 kHz (Svedbrant et al., 2015). Whether there is an ear asymmetry to this decline is unclear as one study found it more pronounced in the right ear (Svedbrant et al., 2015), the other in the left ear (Hederstierna et al., 2010). In addition to peripheral hearing sensitivity, ABR waves latencies were also increased after the start of menopause (Tandon et al., 2001; Trott et al., 2019; Arora et al., 2021). Significant differences in speech reception in noise, as poor performance was found in pre- and post-menopausal women with normal PTA thresholds, suggesting some central hearing loss. The findings of the studies are summarized in Table 4.

**TABLE 4:** Summary of the studies’ findings.

	Static sex differences		Cyclical changes/hormonal fluctuation	
	**Pre-menopausal vs. age-matched men**	**Post-menopausal** **vs. age-matched men**	**Pre-menopausal women**	**Post-menopausal women**
PTA	Women have better performance	Post-menopausal women tend to have steeper decreased hearing sensitivity than men	Better performance during late follicular phase	Fast and rapid decline in hearing in high frequency after the start of menopause.
TEOAEs SOAEs MLSOAEs CEOAEs	Women have better performance		Better performance during late follicular phase	
ABR	Women have better performance		Better performance during early luteal phase reported by most of the studies, however, there was a conflicted result, as it was reported better performance was during late follicular phase.	Longer waves latencies in women between 50–70 years old.
Speech perception	Women have better performance		Better performance during late follicular phase	Poor performance after menopause.
DPOAEs	No significant sex differences in the recordings. The differences are related to the anatomical differences (i.e., the length of the cochlea)		Better performance in late follicular phase Worse recording in the premature ovarian failure (POF) group	
Speech-ABR	Women have better performance		Worse performance during late follicular phase	
40 Hz ASSR	Left-handed women had better performance than left-handed men. No significant differences between right-handed women and men.		Better performance during late follicular phase	
Dichotic testing			Better right ear responses during late follicular phase	Poor responses after menopause.

## 4. Quality of evidence

The quality and risk of bias of the included studies was assessed using Newcastle-Ottawa Scale (NOS). Only four studies were of good quality (Hjelmervik et al., 2012; Melynyte et al., 2018; Carneiro et al., 2019; Zakaria et al., 2019). 27 studies were of fair quality (high risk) (Bowman et al., 2000; Ismail and Thornton, 2003; Serra et al., 2003; Yadav et al., 2003; Dreisbach et al., 2007; Sharashenidze et al., 2008; Wadnerkar et al., 2008; Al-Mana et al., 2010; Hederstierna et al., 2010; Kim et al., 2010; Cowell et al., 2011; Snihur and Hampson, 2011; Svedbrant et al., 2015; Adriztina et al., 2016; Hu and Lau, 2017; Jalaei et al., 2017; Boothalingam et al., 2018; Trott et al., 2019; Zakaria et al., 2019; Karaer and Gorkem, 2020; Arora et al., 2021). Four studies were of poor quality (very high risk) (Tandon et al., 2001; Griskova-Bulanova et al., 2014; Hodgetts et al., 2015; Emami et al., 2018).

The main concern was the method of assessment for hormone levels, as few studies used objective tests such as blood assays and saliva samples. Another factor that affected the quality of the studies which examined the effect of female hormone fluctuation on hearing, was the number of sessions. Only three studies were considered to have a “good” number of sessions for the studied outcomes to occur, as they tested participants in three or four sessions across one menstrual cycle. Finally, most studies included in the review did not have control group. The quality of the studies is summarized in Table 5.

**TABLE 5:** Quality and risk of bias assessment (Newcastle–Ottawa Scale) criteria.

	Selection			Comparability			Outcome		Total quality score	
	**Representativeness of the exposed cohort**	**Selection of the non-exposed cohort**	**Ascertainment of exposure**	**Demonstration that outcome of interest was not present at start of study**	**Comparability of cohorts based on the design or analysis**	**Assessment of outcome**	**Was follow-up long enough for outcomes to occur**	**Adequacy of follow up of cohorts**		
**References**										
Bowman et al., 2000		*		*	*	*			**4**	Fair quality
Tandon et al., 2001	*					*			**2**	Poor quality
Ismail and Thornton, 2003		*		*	*	*			**4**	Fair quality
Dreisbach et al., 2007		*		*	*	*	*		**5**	Fair quality
Sharashenidze et al., 2008	*	*		*		*	*		**5**	Fair quality
Kim et al., 2010	*	*		*	*	*			**5**	Fair quality
Snihur and Hampson, 2011		*		*	*	*			**4**	Fair quality
Jalaei et al., 2017		*		*	*	*	*		**6**	Fair quality
Boothalingam et al., 2018	*	*		*	*	*			**5**	Fair quality
Melynyte et al., 2018	*	*	*	*	*	*	*		**7**	Good quality
Stuart and Kerls, 2018				*	*	*	*		**4**	Fair quality
Zakaria et al., 2019	*	*	*	*	*	*	*		**7**	Good quality
Serra et al., 2003	*		*			*	*	*	**5**	Fair quality
Yadav et al., 2003		*		*	*	*	*	*	**6**	Fair quality
Walpurger et al., 2004			*	*	*	*	*		**5**	Fair quality
Wadnerkar et al., 2008	*	*		*	*	*		*	**6**	Fair quality
Al-Mana et al., 2010	*		*	*		*	*	*	**6**	Fair quality
Hederstierna et al., 2010	*			*		*	*	*	**5**	Fair quality
Cowell et al., 2011	*		*	*		*		*	**5**	Fair quality
Hjelmervik et al., 2012	*	*	*	*	*	*	*		**7**	Good quality
Mann et al., 2012				*	*	*	*		**4**	Fair quality
Griskova-Bulanova et al., 2014			*	*		*			**3**	Poor quality
Upadhayay et al., 2014				*	*	*	*		**4**	Fair quality
Hodgetts et al., 2015			*	*		*			**3**	Poor quality
Svedbrant et al., 2015	*		*	*		*	*		**5**	Fair quality
Adriztina et al., 2016	*			*		*	*	*	**5**	Fair quality
Batta et al., 2017				*	*	*	*	*	**5**	Fair quality
Hu and Lau, 2017				*		*	*	*	**4**	Fair quality
Liu et al., 2017		*	*	*	*	*	*		**6**	Fair quality
Souza et al., 2017		*		*	*	*	*		**5**	Fair quality
Emami et al., 2018				*		*		*	**3**	Poor quality
Carneiro et al., 2019	*	*	*	*	*	*		*	**7**	Good quality
Trott et al., 2019	*	*		*	*			*	**5**	Fair quality
Karaer and Gorkem, 2020		*		*	*	*			**4**	Fair quality
Arora et al., 2021	*	*		*	*			*	**5**	Fair quality

NOS has a total maximum score of 9: Maximum scores 4 in Selection, 2 in Comparability, 3 in Outcome. Studies score from 7–9 have good quality (high quality), 4–6 have fair quality (high risk), and 0–3 have poor quality (very high risk).

The symbol (*) means the point earned in each category.

## 5. Discussion

The aim of the systematic review was to evaluate the current evidence of the differences in auditory function between women and men. In addition, the aim was to review the available literature of the effect of the female sex hormones (i.e., estradiol and progesterone) on fluctuating auditory function in women (i.e., during the menstrual cycle and after menopause).

Eleven studies investigated sex-specific differences in the peripheral and central auditory pathways. They reported that women’s hearing sensitivity was better compared to age-matched men’s, especially at higher frequencies (Kim et al., 2010). Participants age ranged from 15 to 83 years. The mean age of men was 46 years, and the mean age of women was 47 years (which might be considered before menopause). Most women were 30–39 years old (*n* = 242) and 40–49 years old (*n* = 313). The total pooled sample size of this review was large (*n* = 1,116). The consistent finding of the review of better hearing sensitivity of pre-menopausal women compared to men is in agreement with a previous study (*n* = 50,000) that pre-menopausal women have better hearing sensitivity than men (Chung et al., 1983), in particular at higher frequencies (approximately 2–3.5 dB differences at frequencies above 2,000 Hz). Another consistent finding across studies was that hearing sensitivity of pre-menopausal women fluctuates across the menstrual cycle, while men tend to show stable hearing sensitivity. In terms of cyclical changes, PTA thresholds were found to be lowest (i.e., better hearing sensitivity) during the late follicular phase compared to other phases of the cycle (Adriztina et al., 2016; Souza et al., 2017; Emami et al., 2018; Karaer and Gorkem, 2020).

It can be argued that the reduction in hearing sensitivity in older women could be due to normal aging, noise exposure, and ototoxicity. However, the changes in hearing were found to be triggered by the onset of menopause (Hederstierna et al., 2009). And similar changes have also been seen in women with premature ovarian failure (POF). In particular, POF and post-menopausal women groups experienced reduced hearing function compared to normal pre-menopausal women (Karaer and Gorkem, 2020). In addition, while ear asymmetries in hearing loss are inconsistent, better hearing in the right ear could be explained anatomically by the number of estradiol receptors in the inner ear. McFadden (1993) reported that the right inner ear is denser in estradiol receptors than in the left ear. These receptors facilitate the effect of estradiol in the inner ear cells, which may enhance the transition of neural signals from the right ear. Once the level of estradiol is reduced in POF or post-menopause, the reduction in that ear may be particularly noticeable.

Like PTA thresholds, SOAEs and TOAEs were reported to be stronger in women (Ismail and Thornton, 2003; Snihur and Hampson, 2011). The function of OHCs might be better in women when compared to men. This might indicate a fluctuation in the inner ear function because of changes in the female sex hormones.

However, several researchers have suggested that these differences may be due to the anatomical differences in the cochlea’s length rather than related to the biological (Bowman et al., 2000; Dreisbach et al., 2007; Boothalingam et al., 2018). In summary, the results suggest that DPOAEs might not be a useful measure to detect sex differences in the auditory function.

The effect of hormones on central auditory function is less clear. While some consistent evidence exists that pre-menopausal women have better overall central auditory functioning, it is less clear whether there are consistent changes in central auditory function across the menstrual cycle. These effects are exemplified in ABR measures. Specifically, fairly robust evidence exists for sex differences in ABRs at suprathreshold levels, with women generally having better responses than men. When inconsistencies between ABR results were reported, particularly in latencies, a possible explanation may be the variation in session numbers and the use of objective measures for female hormones. The possible contribution of estradiol and progesterone in the central auditory pathways may remain unclear, and whether estradiol or progesterone can improve conduction of auditory neural signals. However, the effect of reduced levels of estradiol in post-menopausal women were found to affect first the central auditory pathway (Trott et al., 2019; Arora et al., 2021). It was found that post-menopausal women with normal hearing sensitivity have longer ABR waves latencies (Hwang et al., 2008; Trott et al., 2019; Arora et al., 2021).

For the studies which included men as control, only fluctuation in hearing was reported in women. Therefore, men may have a stable hearing sensitivity. In other words, due to stable levels of female hormones in men, a stable hearing function was noticed. However, this was reported in only three papers (Hjelmervik et al., 2012; Souza et al., 2017; Carneiro et al., 2019) as both sexes were tested in 3–4 sessions across the cycle, and one study tested both sexes in one session (Liu et al., 2017) and one study tested men in one session only and women in two sessions (Wadnerkar et al., 2008). The variation in the design of papers studied the effect of female sex hormones may cause uncertainty regarding the interpretation of the role of these hormones.

This is the first systematic review that has attempted to address differences in auditory function between the sexes and the possible effect of female sex hormone fluctuation on hearing function. The conclusion of this review is drawn from thirty-three studies. The lack of “good” quality studies makes it challenging to understand the effect of female hormones on hearing in detail. The review highlights the need for objective measures to assess the hormone level at the time of testing. In addition, participants need to be tested in multiple, ideally four or more, sessions throughout the menstrual cycle to detect the effect of hormone changes on hearing, so that errors in test timing can be avoided.

Most of the studies were not controlled, and only three studies included male participants as a control group. All studies conducted in menopausal women did not use any control groups.

In addition, in order to improve objectivity of measures researcher could consider using a blind study design and objective tests such as blood or saliva samples to measure hormones levels. None of the studies included in this review stated the day of the cycle when women were tested. Accurately measuring and reporting this information may help to disambiguate some of the currently inconsistent results.

No studies on the possible effects of hormones overall or fluctuation of estradiol and progesterone on tinnitus or vestibular dysfunction existed highlighting the severe lack of studies on this topic.

In conclusion, there are significant sex differences in peripheral auditory function, particularly PTA threshold, SOAEs, TEOAEs, between pre-menopausal women and age-matched men. In addition, a possible effect of estradiol on peripheral auditory function across the menstrual cycle was reported in most of the included papers. In contrast, the effect of estradiol and progesterone in the central auditory system remains unclear. Whether this difference in results between peripheral and central auditory function reflects a true difference in function or a difference in assessment is currently unclear. PTA is the main tool used in audiology clinics and research, hence more evidence, and importantly more consistent evidence, can accumulate. Tests that assess speech reception in background noise are less frequently used both in research and in the clinic despite their greater usefulness to assess aspects of hearing that are important for everyday listening. This can be an important tool to assess higher regions of the auditory pathway, including cognition. A more frequent use would allow us to build up a more detailed picture of the effect of sex hormones overall and their effect across the menstrual cycle. Finally, it was noticeable how much outcome measures differed between studies, and that the majority of studies did not use an objective test to measure hormones levels. It is recommended for the future studies to include consistent outcome measure which may include audiometric tests such as PTA (including extended high frequencies) and speech audiometry (e.g., SiN).

### 5.1. Deviation from the published protocol

The protocol was restricted to studies with control groups. However, this restriction excluded many studies that investigated the fluctuation of female hormones and changes in hearing sensitivity. These studies used objective hormonal tests and a greater number of sessions. Therefore, papers without control groups were included in the review, but their qualities were affected by that.

## Author contributions

NA wrote the manuscript with input from all authors. AH, KM, and KK edited the manuscript. All authors discussed the results and contributed to the final version of the manuscript.
