# Supplementary material for: Sex differences and the effect of female sex hormones on auditory function: a systematic review
Source: Front Hum Neurosci. 2023 Apr 21;17:1077409. doi: 10.3389/fnhum.2023.1077409 (PMC10161575; doi:10.3389/fnhum.2023.1077409)
Supplement: Supplementary file 1 [file Data_Sheet_1.DOCX]

Supplementary Material

# Supplementary Material

(("Hearing"[Mesh] OR "Hearing"[tw] OR "Auditory Perception"[mesh] OR "Auditory Perception"[tw] OR "auditory function"[tw] OR "auditory sensitivity"[tw] OR "Speech Perception"[Mesh] OR "speech perception"[tw] OR "Tinnitus"[Mesh] OR "tinnitus"[tw] OR "hearing level"[tw] OR "hearing levels"[tw] OR "auditory level"[tw] OR "auditory levels"[tw] OR "ringing" OR "buzzing"[tw] OR "Speech-in-noise"[tw]) AND ("Estrogens"[Mesh] OR "Estrogens"[Pharmacological Action] OR "oestrogen"[tw] OR "oestrogens"[tw] OR "estrogen"[tw] OR "estrogens"[tw] OR "2,3-bis(3'-hydroxybenzyl)butane-1,4-diol"[supplementary concept] OR "2,3-bis(3'-hydroxybenzyl)butyrolactone"[supplementary concept] OR "4-octylphenol"[supplementary concept] OR "8-prenylnaringenin"[supplementary concept] OR "biochanin A"[supplementary concept] OR "bisphenol A"[supplementary concept] OR "Chlorotrianisene"[mesh] OR "Coumestrol"[mesh] OR "daidzein"[supplementary concept] OR "Dienestrol"[mesh] OR "Diethylstilbestrol"[mesh] OR "diethylstilbestrol dipropionate"[supplementary concept] OR "Epimestrol"[mesh] OR "Equol"[mesh] OR "Estradiol"[mesh] OR "estradiol enanthate"[supplementary concept] OR "Estrogenic Steroids, Alkylated"[mesh] OR "Estrogens, Conjugated (USP)"[mesh] OR "estrogens, conjugated synthetic A"[supplementary concept] OR "estrogens, conjugated synthetic B"[supplementary concept] OR "Estrogens, Esterified (USP)"[mesh] OR

OR "Oestradiol"[tw] OR "Estradiol"[tw] OR "estradiol enanthate"[tw] OR "Estrogenic Steroids, Alkylated"[tw] OR "Estrogens, Conjugated (USP)"[tw] OR "estrogens, conjugated synthetic A"[tw] OR "estrogens, conjugated synthetic B"[tw] OR "Estrogens, Esterified (USP)"[tw] OR "Estrone"[tw] OR "Ethinyl Estradiol"[tw] OR "formononetin"[tw] OR "Genistein"[tw] OR "glycitein"[tw] OR "glycitin"[tw] OR "Hexestrol"[tw] OR "Infecundin"[tw] OR "Mestranol"[tw] OR "nylestriol"[tw] OR "o,p'-DDT"[tw] OR "O-desmethylangolensin"[tw] OR "polyestradiol phosphate"[tw] OR "Quinestrol"[tw] OR "secoisolariciresinol"[tw] OR "Zearalenone"[tw] OR "Zeranol"[tw] OR "Estradiol"[Mesh] OR "estradiol"[tw] OR "oestradiol"[tw] OR "Progynon Depot"[tw] OR "Delestrogen"[tw] OR "Ovocyclin"[tw] OR "sex hormone"[tw] OR "sex hormones"[tw] OR "Gonadal Steroid Hormones"[Mesh] OR "Gonadal Steroid Hormone"[tw] OR "Gonadal Steroid Hormones"[tw] OR "Androstane-3,17-diol"[tw] OR "Androstenediol"[tw] OR "Androstenedione"[tw] OR "Androsterone"[tw] OR "Dehydroepiandrosterone"[tw] OR "Dihydrotestosterone"[tw] OR "Equilenin"[tw] OR "Equilin"[tw] OR "Estradiol"[tw] OR "Estriol"[tw] OR "Estrogenic Steroids"[tw] OR "Estrone"[tw] OR "Etiocholanolone"[tw] OR "Pregnenolone"[tw] OR "Progesterone"[tw] OR "20-alpha-Dihydroprogesterone"[tw] OR "Algestone"[tw] OR "Algestone Acetophenide"[tw] OR "Hydroxyprogesterones"[tw] OR "17-alpha-Hydroxyprogesterone"[tw] OR "Medroxyprogesterone"[tw] OR "Progesterone Congeners"[tw] OR "Testosterone"[tw] OR "Testosterone Congeners"[tw] OR "steroid hormone"[tw] OR "steroid hormones"[tw] OR "Steroids"[Mesh] OR "Steroid"[tw] OR "Steroids"[tw] OR "17-Ketosteroids"[tw] OR "17-Ketosteroid"[tw] OR "Androstanes"[tw] OR "Androstane"[tw] OR "Androstanols"[tw] OR "Androstanol"[tw] OR "Androstenes"[tw] OR "Androstene"[tw] OR "Azasteroids"[tw] OR "Estriol"[Mesh] OR "Estriol"[tw] OR "Ovestin"[tw] OR "Epiestriol"[tw] OR "Estetrol"[tw]))
